# The psychological impact of vernal keratoconjunctivitis on families: An investigation on quality of life and psychological wellbeing

**DOI:** 10.1111/pai.70141

**Published:** 2025-07-03

**Authors:** Ludovica Natali, Valentina Cardi, Fabiano Cavarzeran, Andrea Leonardi

**Affiliations:** ^1^ Department of General Psychology University of Padova Padova Italy; ^2^ Department of Neuroscience, Ophthalmology Unit University of Padova Padova Italy

**Keywords:** anxiety, caregivers, depression, psychological wellbeing, quality of life, vernal keratoconjunctivitis

## Abstract

**Background:**

Vernal keratoconjunctivitis (VKC) is a chronic allergic eye disease that affects children and adolescents. While its physical burden is well documented, little research has explored its psychological impact on patients and their families. This study examined the impact of VKC on quality of life (QoL) and psychological wellbeing in children (as reported by their parents) and their parents.

**Methods:**

Forty parents of children with VKC (aged 4–17 years) attending the Ocular Allergy and Immunology Service at the University of Padova completed online questionnaires. Measures included parental depressive (PHQ‐9) and anxiety symptoms (GAD‐7), perceived social support, family QoL (FROM‐16), child emotional and behavioral difficulties (SDQ), and the impact of VKC on child QoL (QUICK). Clinical data on VKC severity and treatment history were also collected.

**Results:**

One‐third of parents reported mild depressive symptoms, and 53% had mild to severe anxiety. Higher parental distress was linked to greater family QoL disruption, while greater perceived support was associated with less disruption. Most children showed emotional and behavioral difficulties within the normal range. However, those with more emotional difficulties and the perennial form of VKC experienced greater QoL impairment. Interestingly, higher QoL disruption in children correlated with more frequent prosocial behaviors.

**Conclusion:**

These findings highlight the psychological impact of VKC on both children and parents. Integrated care approaches that address both physical and psychological aspects of the disease are essential to improve outcomes for affected families.

**Key message**

This study highlights the profound impact of vernal keratoconjunctivitis (VKC) on the quality of life and psychological wellbeing of children and their parents. Using validated measures, we reveal the psychological distress and family burden associated with this rare yet debilitating condition. Our findings emphasize the need for a holistic approach to VKC management, addressing not only clinical symptoms but also the emotional and social challenges faced by families. These insights are critical for healthcare providers and researchers aiming to improve care for VKC patients and their families.

## 1 INTRODUCTION

Vernal keratoconjunctivitis (VKC) is a severe, chronic type 2 ocular allergic disease that primarily affects children and adolescents. It causes prolonged ocular symptoms that last for several months per year and may persist for years.^1^, ^2^ The prevalence of VKC is estimated at less than 2 in 10,000 in the United States and 3.2 in 10,000 in Europe, with Italy being among the countries with the highest prevalence.^3^, ^4^, ^5^ VKC is more common in tropical areas; for instance, a prevalence of 11% was reported in an Ethiopian school community, and 36.4% among children who missed school in Rwanda.^5^

Patients with VKC experience intense itching, photophobia, tearing, and mucous discharge which disrupt their social and school lives, causing psychological distress for both themselves and their families.^6^ While the psychological implications of other allergic diseases, such as asthma and urticaria, are well‐documented,^7^ limited research has explored the specific psychological consequences of VKC.^8^, ^9^ The questionnaire “Quality of Life in Children with Vernal Keratoconjunctivitis” (QUICK) was developed to assess the physical, psychosocial, and practical implications of the illness on patients' quality of life (QoL).^10^ This tool has been used in clinical trials to evaluate the efficacy of treatments, such as topical cyclosporine.^11^ The QUICK has proven to be a reliable measure of the child's subjective experience of the disease, showing strong correlations with subjective symptom measures like visual analogue scales (VAS), but weaker associations with objective clinical parameters.^8^, ^9^, ^10^, ^12^ This suggests that the QUICK offers unique insights into how VKC affects daily life and wellbeing, beyond clinical severity.

Chronic pediatric illnesses, such as VKC, can significantly impact not only the patients but also the psychological wellbeing of their families.^13^, ^14^ A meta‐analysis revealed that 57% of parents caring for chronically ill children met clinical thresholds for anxiety, compared to 38% of parents of healthy children. Similarly, 35% of these parents met criteria for clinical depression, compared to 19% in controls.^13^ These findings underscore the importance of addressing the psychological wellbeing of both young patients and their caregivers in managing chronic conditions. Therefore, the aim of this study was to investigate the impact of VKC on QoL and psychological wellbeing of both children (as reported by their parents) and their parents.

## 2 METHODS

### 2.1 Participants

Participants were parents of children aged 4–17 years old and with a diagnosis of VKC (based on clinical history, signs, and symptoms)^11^, ^12^, ^15^, ^16^ who attended the Ocular Allergy and Immunology Service at the University Hospital of Padova from March 2023 to October 2023. Participants were recruited during routine clinical practice and asked to complete the study's questionnaires on the Qualtrics XM platform. The study complied with the Declaration of Helsinki and was approved by the ethical committee of the University of Padova (reference number: 1100‐b). Written informed consent was provided by all participants.

### 2.2 Clinical assessment of VKC in children

Clinical and demographic information was collected for all children affected by VKC whose parents had agreed to participate in the study. This information consisted of data on age, gender, age of onset, duration, family history of allergy, disease phenotype (tarsal, limbal, or mixed), positive or negative IgE testing, current therapy with antiallergic eyedrops or topical immunomodulators, and the use of topical corticosteroids or topical immunomodulators. Patients with moderate to severe symptoms besides immunomodulation therapy were considered refractory VKC patients.^17^ A conventional slit‐lamp examination was performed. A VKC activity score, in accordance with the Bonini clinical grading, was given to each patient^11^, ^12^, ^15^, ^16^, ranging from grade 0 = quiescent, to 1 = mild, 2 = moderate, 3 = severe, and 4 = very severe.

### 2.3 Psychological measures

Participants (i.e., parents of children with VKC) answered a demographic questionnaire including questions about themselves (e.g., age, gender, years of education, familiarity with chronic illness or psychological difficulties) and their child (e.g., age, illness duration). Participants also completed the following questionnaires:

*Patient Health Questionnaire‐9 (PHQ‐9)*,^18^, ^19^ a 9‐item questionnaire to assess depressive symptoms. The total score ranges from 0 to 27, and higher scores indicate greater symptom severity. Total scores of 5, 10, 15, and 20 represent cut‐offs for mild, moderate, moderately severe, and severe depression, respectively. The Cronbach's alpha of the total score in this study was 0.8.

*General Anxiety Disorder‐7 (GAD‐7)*,^20^ a 7‐item questionnaire to assess anxiety symptoms over the past 2 weeks. The total score ranges from 0 to 21, and higher scores indicate greater symptom severity. Total scores of 5, 10, and 15 represent cut‐offs for mild, moderate, and severe anxiety, respectively. The Cronbach's alpha of the total score in this study was 0.9.

*Family Reported Outcome Measure (FROM‐16)* ^21^ to assess the impact of VKC on family quality of life in the emotional, personal, and social life domains. The total score ranges from 0 to 32, with higher scores indicating a greater impact of the disease on family members. Total scores of 2, 9, 17, and 26 represent cut‐offs for small, moderate, very large, and extremely large effects on family QoL, respectively. In this study, the Cronbach's alpha of the emotional domain was 0.8; the Cronbach's alpha of the personal and social life domain and the total score was 0.9.

*Perceived social support*. To assess perceived social support, two ad‐hoc questions were developed by the research team. These questions aimed to evaluate the extent to which parents felt supported by family members and/or friends in two specific areas: (1) assisting their child in managing the symptoms (i.e., “How supported do you feel by other family members and/or friends in helping your child manage the symptoms of their illness?”), and (2) addressing their own concerns or fears related to their child's illness (i.e., “How supported do you feel by other family members and/or friends regarding your concerns or fears about your child's illness?”). Participants rated their level of perceived support for each question on a 4‐point Likert scale ranging from 0 (“not at all”) to 3 (“Extremely”).

*Strengths and Difficulties Questionnaire (SDQ)* ^22^, ^23^, ^24^ to assess children's behavioral, emotional, and interpersonal difficulties and strengths as perceived by their parents. In this study, the informant‐rated version for parents of 4–17 years old was used. The SDQ included five scales (i.e., emotional difficulties, conduct difficulties, hyperactivity, peer difficulties, and prosocial scale). The SDQ allows obtaining a total score (by summing scores from all the scales except the prosocial scale). Total scores of 14, 17, and 20 represent cut‐offs for slightly raised, high, and very high total difficulties, respectively. For a detailed description of the cut‐offs for each subscale, please refer to the measure's website (www.sdqinfo.org/py/sdqinfo/c0.py) In this study, the Cronbach's alpha was 0.8 for the emotional difficulties scale, the hyperactivity scale, and the prosocial scale, and 0.7 for the conduct difficulties scale.

*Quality of Life in Children with Vernal Keratoconjunctivitis (QUICK)* ^10^, ^25^ to assess the impact of VKC on children's quality of life, as reported by their parents. The questionnaire evaluates the frequency of clinical symptoms of the disease and their impact on daily activities. In this study, the Cronbach's alpha for the symptoms' domain, the daily activities domain, and the total score was 0.9.

### 2.4 Statistical analyses

All statistical analyses were performed with JASP (Version 0.19, JASP Team, 2024). Data are reported as mean ± standard deviation (SD) or frequency (%). Spearman's correlations were performed to explore the associations between children's clinical symptoms of the disease, parents' psychological wellbeing (i.e., depressive and anxiety symptoms), children's psychological wellbeing (i.e., strengths and difficulties), impact of the illness on children (i.e., in term of perceived symptoms severity and impact on daily activities) and parents' quality of life (i.e., its impact on emotional and social domains), parents' perceived social support in assisting their child in managing the symptoms and addressing their own concerns/fears related to their child's illness, and comorbidities.

## 3 RESULTS

### 3.1 Socio‐demographic and clinical characteristics

A total of 40 parents of 40 children with a diagnosis of VKC completed the study's measures. The socio‐demographic and psychological characteristics of parents and their children are presented in Tables 1, 2, 3. Parents' mean age was 44.5 ± 7.0 years. Most were of female gender, married, and of Italian nationality. The majority had a high school diploma and were employed. Most of them reported spending no more than 7 hours per week taking care of their child's illness. The majority of participants reported feeling “much” (*n* = 8, 20%) to “extremely” supported (*n* = 15, 37.5%) in helping their child manage VKC symptoms; 11 reported feeling “somewhat” supported (27.5%) and 6 not feeling supported (15.0%). The majority of participants also reported feeling “much” (*n* = 12, 30%) to “extremely” supported (*n* = 9, 22.5%) in addressing their own concerns/fears related to their child's illness; 15 reported feeling “somewhat” supported (37.5%) and 4 not feeling supported (10.0%).

**TABLE 1:** Parents' socio‐demographic characteristics expressed as means (standard deviations SD; range) or frequencies (%). *N* = 40.

Variable	Statistics
Age	44.5 (7.0; 29–57)
Sex (Female vs. Male)	37 (92.5%)
Nationality (Italian vs. other)	38 (95.0%)
First language (Italian vs. other)	37 (92.5%)
Level of education	
Inferior to Diploma	1 (2.5%)
Diploma	24 (60.0%)
Bachelor's Degree	3 (7.5%)
Master's Degree	10 (25.0%)
PhD	1 (2.5%)
Other title (e.g., conservatory, academic of fine arts)	1 (2.5%)
Employment	
Full‐time worker	19 (47.5%)
Part‐time worker	14 (35.0%)
Self‐employed	2 (5.0%)
Homemaker	3 (7.5%)
Unemployed (actively seeking employment)	2 (5.0%)
Marital status	
Single	4 (10.0%)
Cohabiting	2 (5.0%)
Married	32 (80.0%)
Separated	1 (2.5%)
Divorced	1 (2.5%)
Number of children	
1	8 (20.0%)
2	28 (70.0%)
3	3 (7.5%)
4	1 (2.5%)
Time spent supporting the child in symptoms management (including medication adherence)	
0–7 h/week	33 (82.5%)
8–14 h/week	6 (15.0%)
>21 h/week	1 (2.5%)

Children were mostly males (70%) with a mean age of 10.3 ± 3.5 (Table 3). The mean time since diagnosis was 42.4 ± 31.7 months, while the average time since symptoms onset was 58.9 ± 36.3 months. At the time of the ophthalmological evaluation, 16 patients (40%) had a mildly severe disease, 16 (40%) had a moderately severe disease, 6 (15%) had a severe disease, and 2 (5%) had a very severe disease. The majority of patients (67.5%) had a limbal form, 5% had tarsal, and 27.5% had the mixed form of VKC. The large majority of patients (92.5%) were affected by a seasonal VKC (from March to October) but only 50% of patients were IgE‐positive to environmental allergens. The majority of patients (77.5%) were treated with on‐label topical CsA 0.1%, and 9 were treated with the combination of olopatadine eye drops and spaglumic acid 6%. Irrespective of the ongoing treatment, 21 had to use at least one course of topical dexamethasone 0.1% for 3 consecutive days as a rescue medication, and 9 (22.5%) were refractory to therapy.

### 3.2 Psychological wellbeing

According to the PHQ‐9 scores, the majority of parents reported depressive symptoms within the normal range (*n* = 25, 62.5%). However, a third of the sample (*N* = 12; 30.0%) experienced mild depressive symptoms, while one parent (2.5%) fell within the moderate range and two (5.0%) in the moderately severe range.^18^, ^26^ Similarly, according to the GAD‐7 scores, most parents (*n* = 19, 47.5%) reported minimal anxiety levels. Thirteen parents (32.5%) experienced mild anxiety, seven (17.5%) reported moderate anxiety, and one parent (2.5%) experienced severe anxiety.^26^ Most parents rated their children's difficulties within the normal range (*n* = 30, 75.0%) on the SDQ. Four parents (10.0%) reported slightly raised difficulties, two (5.0%) indicated high levels, and four (10.0%) indicated very high levels^22^ (Table 2). Specifically (Table S1), regarding emotional difficulties, 25 parents (62.5%) rated their child's difficulties as average; 5 (12.5%) as slightly raised; 4 (10%) parents as high, and 6 (15%) very high. Regarding conduct problems, 27 parents (67.5%) reported average scores, 6 (15%) slightly raised, 5 (12.5%) high scores, and 2 (2%) very high scores. Regarding hyperactivity, 35 parents (87.5%) reported average scores, 2 (5%) reported slightly higher scores, another 2 (5%) high scores, and 1 (2.5%) very high score. For peer difficulties, most parents reported average scores (*n* = 28, 70%), 5 (12.5%) slightly raised, 4 (10%) high scores, and 3 (7.5%) very high scores. Finally, most parents rated their child's prosocial behavior as average (*n* = 25, 62.5%), 5 (12.5%) reported slightly lower scores, 6 (15%) low scores, and 4 (10%) very low scores.

**TABLE 2:** Parents' psychological characteristics expressed as means (standard deviations SD; and range) or frequencies (%). *N* = 40.

Variable	Statistics
Perceived support from family and friends in managing the child's symptoms	
Overall score	1.80 (1.11; 0–3)
Not feeling	6 (15.0%)
Somewhat	11 (27.5%)
Much	8 (20.0%)
Extremely	15 (37.5%)
Perceived support from family and friends regarding concerns about the child's illness	
Overall score	1.65 (0.95; 0–3)
Not feeling	4 (10.0%)
Somewhat	15 (37.5%)
Much	12 (30.0%)
Extremely	9 (22.5%)
Family Reported Outcome Measures—FROM‐16	
Emotional	4.30 (2.87; 0–11)
Personal and social life	3.53 (3.76; 0–18)
Total score	7.83 (6.25; 1–29)
None	3 (7.5%)
Small	23 (57.5%)
Moderate	10 (25.0%)
Very large	3 (7.5%)
Extremely large	1 (2.5%)
Patient Health Questionnaire—PHQ‐9	
Overall score	4.03 (4.03; 0–18)
Minimal	25 (62.5%)
Mild	12 (30.0%)
Moderate	1 (2.5%)
Moderately severe	2 (5.0%)
General Anxiety Disorder—GAD‐7	
Overall score	5.73 (4.54; 0–21)
Minimal	19 (47.5%)
Mild	13 (32.5%)
Moderate	7 (17.5%)
Severe	1 (2.5%)
Strengths and Difficulties Questionnaire—SDQ‐25	
Emotional symptoms	3.10 (2.45; 0–10)
Conduct problems	1.80 (1.70; 0–7)
Hyperactivity/inattention	2.78 (2.60; 0–10)
Peer relationship problems	1.68 (1.94; 0–7)
Prosocial behaviors	7.63 (2.27; 1–10)
Total difficulties score	9.35 (7.18; 0–28)
Normal	30 (75.0%)
Slight	4 (10.0%)
High	2 (5.0%)
Very high	4 (10.0%)

**TABLE 3:** Patients' socio‐demographic and psychological characteristics expressed as mean (standard deviation SD; range) or frequencies (%). *N* = 40.

Variable	Statistics
Age (years)	10.3 (3.5; 4–17)
Sex (Male vs. Female)	28 (70.0%)
Time since first diagnosis (months)	42.5 (31.7; 1–108)
Illness duration (months)	59.0 (36.3; 3–156)
Illness's severity score	
1	16 (40.0%)
2	16 (40.0%)
3	6 (15.0%)
4	2 (5.0%)
Illness phenotype	
1	27 (67.5%)
2	2 (5.0%)
3	11 (27.5%)
Seasonality of the illness (Yes vs. No)	37 (92.5%)
Receiving treatment for the illness (Yes vs. No)	9 (22.5%)
Comorbidities	
None	24 (60.0%)
Rhinitis	11 (27.5%)
Asthma	3 (7.5%)
Dermatitis	2 (5.0%)
Quality of Life in Children with Vernal Keratoconjunctivitis – QUICK	
Symptoms	46.9 (21.6; 8.3–91.7)
Daily activities	25.3 (27.9; 0–100)
Total score	41.5 (22.1; 9.4–90.6)

### 3.3 The impact of VKC on QoL

Based on the FROM‐16, most parents reported that their child's disease had a small (*n* = 23, 57.5%) or moderate (*n* = 10, 25.0%) impact on family QoL. Three parents (7.5%) indicated no effect, while another three (7.5%) reported a very large effect. Only one parent (2.5%) described the impact as extremely large.^27^

Parents reports on perceived symptom severity and impact of VKC on their child's daily activities, as measured by the QUICK, were consistent with prior research^9^.

**FIGURE 1:**
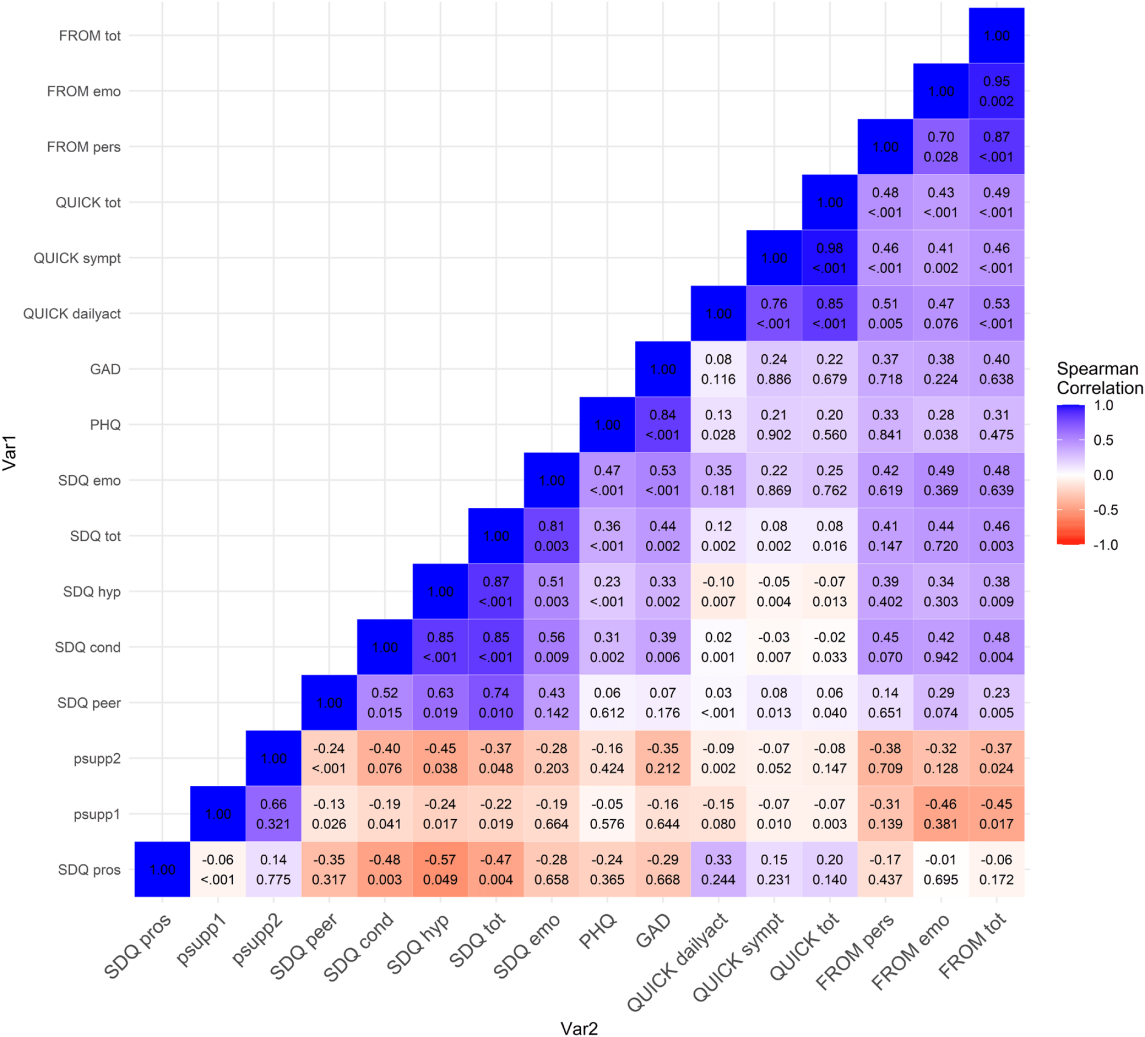
Spearman's rho heatmap displaying pairwise correlations between psychological distress and disruption of quality of life due to keratoconjunctivitis in children with VKC and their parents. Blue squares indicate positive correlations, red squares indicate negative correlations. More saturated squares indicate stronger associations. Within each square, the top value shows the Spearman's correlation coefficient, while the bottom value shows the corresponding *p*‐value. *N* = 40. FROM, Family Reported Outcome Measures‐16; FROM tot, Total score; FROM emo, Emotional domain; FROM pers, Personal and social life domain; PHQ, Patient Health Questionnaire‐9; GAD, General Anxiety Disorder‐7; SDQ, Strengths and Difficulties Questionnaire; SDQ emo, Emotional difficulties scale; SDQ hyp, Hyperactivity scale; SDQ cond, Conduct difficulties scale; SDQ peer, Peer difficulties scale; SDQ pros, Prosocial scale; SDQ tot, Total score; QUICK, Quality of Life in Children with Vernal Keratoconjunctivitis; QUICK sympt, Symptoms scale; QUICK dailyact, Daily activities subscale. Psupp1, Perceived support from family and friends in managing the child's symptoms. Psupp2, Perceived support from family and friends in addressing fears and concerns related to the child's illness.

Correlational analyses showed that greater QoL disruption caused by VKC in children was significantly associated with greater QoL disruption in parents (rho = 0.48, *p* = .002), as indicated by the significant correlations across all subscales of the QUICK and FROM‐16 (Figure 1).

### 3.4 QoL, psychological well‐being and perceived support among parents

Parents reporting greater disruption of family QoL due to the disease (FROM‐16 – Total score) also experienced greater psychological distress (i.e., depressive symptoms, PHQ‐9, rho = 0.31, *p* = .048; anxiety symptoms, GAD‐7, rho = 0.40, *p* = .010; Figure 1). Greater difficulties in the emotional domain specifically (FROM‐16 – Emotional domain) were related to increased anxiety (GAD‐7, rho = 0.38, *p* = .015), whereas greater difficulties in the personal and social life domains (FROM‐16 – Personal and social life domain) were associated with greater anxiety (GAD‐7, rho = 0.37, *p* = .019) and also with depressive symptoms (PHQ‐9, rho = 0.33, *p* = .038). Parents who reported less disruption in family QoL (FROM– Total score) reported greater perceived support in caring for their child's illness (rho = −0.45, *p* = .004) and in managing illness‐related worries (rho = −0.37, *p* = .019). Those feeling more supported in managing illness‐related worries also reported less anxiety (GAD‐7, rho = −0.35, *p* = .026).

### 3.5 QoL and psychological well‐being among children with VKC

Parents reports of their children's QoL as impacted by the disorder indicated an association between greater disruption of daily activities (QUICK – Daily activities subscale) and more emotional problems (SDQ – Emotional difficulties scale) (rho = 0.35, *p* = .028). An interesting correlation was also found between greater disruption of daily activities (QUICK – Daily activities subscale) and a higher frequency of prosocial behaviors observed in children by their parents (SDQ – Prosocial scale) (rho = 0.33, *p* = .038) (Figure 1).

QoL in children, as measured by the QUICK, was not correlated with illness's clinical parameters, including the clinical severity score (*rho* = 0.14, *p* = .383), disease phenotype (rho = −0.04, *p* = .786), response to treatment (rho = 0.18, *p* = .255), and IgE‐sensitization (rho = −0.08, *p* = .623) and the presence of comorbidities (rho = −0.02, *p* = .914). However, the perennial condition of the disease was associated with perceived symptoms severity (QUICK – symptoms subscale, rho = 0.36, *p =* .022) and their impact on daily activities (QUICK – daily activities subscale, rho = 0.40, *p =* .011). Symptoms severity (QUICK – symptoms subscale) was also associated with the use of topical corticosteroids as rescue medication (rho = 0.31, *p =* .050).

## 4 DISCUSSION

This study highlights the impact of VKC on the QoL and psychological well‐being of both children (as reported by their parents) and their parents. To our knowledge, this is the first study to account for psychological variables and QoL not only in patients with VKC but also among their parents. Overall, our findings underscore the interplay between psychological well‐being and QoL impairments in both parents and children, emphasizing the crucial role of social support.

Parents of children with VKC generally reported depressive and anxiety symptoms within the normal range. However, approximately 30% of them reported mild depressive symptoms, and a smaller proportion met the criteria for moderate to moderately severe depression. Anxiety symptoms were more frequent, with over half of the sample reporting levels ranging from mild to severe. These rates are in line with those observed in parents of children with other chronic pediatric conditions.^13^, ^14^ Although the majority of parents perceived the disease as having only a small to moderate impact on family QoL, those experiencing greater psychological distress also reported greater disruption in family QoL. Interestingly, most parents also reported spending relatively little time managing their child's symptoms. These findings suggest that the psychological burden experienced by parents may be driven not solely by caregiving demands, but also by the emotional toll of witnessing their child's discomfort.

Social support emerged as a protective factor. While parents generally reported high levels of social support, particularly regarding practical aspects of care, they perceived less support in relation to concerns and fears regarding their child's illness. Perceived social support was inversely associated with disruption in family QoL. Parents who felt most supported in addressing their concerns reported also lower levels of anxiety. These results are consistent with a broader literature highlighting the buffering role of social support in contexts of psychological stress.^28^, ^29^

Children with VKC were reported to have emotional and behavioral difficulties predominantly within the normal range, as assessed by the SDQ. However, a subgroup displayed elevated difficulties, with emotional problems being the most frequently reported area of concern. QUICK scores indicated an impact of the disease on children's QoL consistent with prior reports.^18^, ^30^ Greater impairment in daily functioning was associated with greater emotional difficulties. Interestingly, a stronger impact of VKC on daily activities was also linked to greater frequency of prosocial behaviors. One possible interpretation is that children with VKC may develop enhanced social sensitivity as a way to cope with illness‐related limitations.^31^ Alternatively, children who are more socially attuned may be more affected by restrictions on daily life, resulting in a greater perceived burden. These hypotheses remain speculative and should be further investigated in future studies. Clinical parameters, such as disease phenotype, type of therapy, comorbidities, or IgE sensitization were not significantly associated with disruption in children's QoL. However, seasonality emerged as a significant factor. Parents of children with perennial VKC reported more severe symptoms and greater interference with daily activities. In these cases, symptoms persist through the year, restricting activities even during winter months. This suggests that QoL impairments may be more closely linked to subjective symptom burden and its persistence across time, rather than to objective clinical parameters. These findings are in line with previous research showing that a single clinical parameter does not fully account for QoL disruption in VKC.^25^

This study has several limitations that should be considered when interpreting the findings. First, the cross‐sectional design does not allow for conclusions about causality between VKC severity, psychological wellbeing, and QoL outcomes. It is also possible that pre‐existing vulnerabilities in parents – such as a history of psychological difficulties or limited social support – may have contributed to the observed associations. Future longitudinal studies are needed to examine the directionality of effects and how fluctuations in disease severity over time influence, and are influenced by, changes in psychological wellbeing and QoL in both children and their parents. This would allow to test for potential bidirectional effects and identify critical periods for psychological interventions. Second, the questionnaires completed by parents did not explicitly focus on the impact of caring for a child with VKC. Future research could explore how caregiving itself contributes to the psychological difficulties experienced by parents. Third, perceived social support was assessed using two ad‐hoc questions, rather than a standardized measure. While this allowed for a brief and context‐specific assessment, the use of validated instruments such as the Multidimensional Scale of Perceived Social Support (MSPSS)^32^ would improve reliability and facilitate comparisons across studies. Fourth, child outcomes were based on parent‐reported measures, which may have introduced reporting bias or inaccuracies. Finally, the small sample size may have limited statistical power, particularly for detecting small to moderate effects. The sample was also predominantly composed of mothers, which reflects typical caregiving roles but limits generalizability to fathers or other caregivers.^33^ Cultural gender norms might have also influenced how psychological distress and social support were perceived and reported. More diverse caregiver samples are needed to clarify these effects.

In conclusion, this study highlights the multifaceted impact of VKC on both children and their parents, emphasizing the significant psychological burden associated with managing this chronic condition. These findings underscore the need for a more integrated model of care, in which psychological well‐being is considered alongside clinical management. Psychoeducational programs specifically developed for families affected by VKC may represent a useful resource to increase awareness, improve disease management, and reduce distress. Embedding psychological support within pediatric ophthalmology services may further enhance access to care. This could include routine screening for psychological difficulties, early identification of families in need, and the provision of tailored support delivered by professionals familiar with the challenges of chronic pediatric illness. Longitudinal research is warranted to inform the development of such interventions and to identify when and for whom psychological support is most beneficial.

## AUTHOR CONTRIBUTIONS

**Ludovica Natali:** Conceptualization; writing – original draft; investigation; methodology; validation; writing – review and editing; formal analysis. **Valentina Cardi:** Conceptualization; validation; methodology; writing – review and editing; supervision; resources. **Fabiano Cavarzeran:** Methodology; validation; software; formal analysis; data curation. **Andrea Leonardi:** Conceptualization; investigation; writing – original draft; methodology; validation; writing – review and editing; project administration; supervision; resources.

## FUNDING INFORMATION

None.

## CONFLICT OF INTEREST STATEMENT

Ludovica Natali: none. Valentina Cardi: none. Fabiano Cavarzeran: none. Andrea Leonardi is a consultant for Alcon, Dompè, FAES Farma, FIDIA, Santen Pharmaceutical Co. Ltd., Laboratoires Théa, and SIFI.

## PEER REVIEW

The peer review history for this article is available at https://www.webofscience.com/api/gateway/wos/peer‐review/10.1111/pai.70141.

## Supporting information


**Table S1.** Strengths and Difficulties Questionnaire (SDQ‐25) subscales.
