# Supplementary material for: The psychological impact of vernal keratoconjunctivitis on families: An investigation on quality of life and psychological wellbeing
Source: Pediatr Allergy Immunol. 2025 Jul 3;36(7):e70141. doi: 10.1111/pai.70141 (PMC12231923; doi:10.1111/pai.70141)
Supplement: Supplementary file 1 — Table S1. Strengths and Difficulties Questionnaire (SDQ‐25) subscales. [file PAI-36-e70141-s001.docx]

**Supplementary Table S1. Strengths and Difficulties Questionnaire (SDQ-25) subscales**

| **Strengths and Difficulties Questionnaire – SDQ-25** | **Frequency (%)** |
| --- | --- |
| *Emotional difficulties (Score: 3.10±2.45, range 0-10)* |  |
| As average | 25 (62.5%) |
| Slightly raised | 5 (12.5%) |
| High | 4 (10.0%) |
| Very high | 6 (15.0%) |
| *Conduct problems (Score: 1.80±1.70, range 0-7)* |  |
| As average | 27 (67.5%) |
| Slightly raised | 6 (15.0%) |
| High | 5 (12.5%) |
| Very high | 2 (2.0%) |
| *Hyperactivity/Inattention (Score: 2.78±2.60, range 0-10)* |  |
| As average | 35 (87.5%) |
| Slightly higher | 2 (5.0%) |
| High | 2 (5.0%) |
| Very high | 1 (2.5%) |
| *Peer relationship problems (Score: 1.68±1.94, range 0-7)* |  |
| As average | 28 (70.0%) |
| Slightly raised | 5 (12.5%) |
| High | 4 (10.0%) |
| Very high | 3 (7.5%) |
| *Prosocial behavior (Score: 7.63±2.27, range 1-10)* |  |
| As average | 25 (62.5%) |
| Slightly lower | 5 (12.5%) |
| Low | 6 (15.0%) |
| Very low | 4 (10.0%) |
